# Addiction of Cancer Stem Cells to MUC1-C in Triple-Negative Breast Cancer Progression

**DOI:** 10.3390/ijms23158219

**Published:** 2022-07-26

**Authors:** Nami Yamashita, Donald Kufe

**Affiliations:** Dana-Farber Cancer Institute, Harvard Medical School, Boston, MA 02215, USA

**Keywords:** MUC1-C, TNBC, CSC, DNA damage resistance, immune evasion

## Abstract

Triple-negative breast cancer (TNBC) is an aggressive malignancy with limited treatment options. TNBC progression is associated with expansion of cancer stem cells (CSCs). Few insights are available regarding druggable targets that drive the TNBC CSC state. This review summarizes the literature on TNBC CSCs and the compelling evidence that they are addicted to the MUC1-C transmembrane protein. In normal epithelia, MUC1-C is activated by loss of homeostasis and induces reversible wound-healing responses of inflammation and repair. However, in settings of chronic inflammation, MUC1-C promotes carcinogenesis. MUC1-C induces EMT, epigenetic reprogramming and chromatin remodeling in TNBC CSCs, which are dependent on MUC1-C for self-renewal and tumorigenicity. MUC1-C-induced lineage plasticity in TNBC CSCs confers DNA damage resistance and immune evasion by chronic activation of inflammatory pathways and global changes in chromatin architecture. Of therapeutic significance, an antibody generated against the MUC1-C extracellular domain has been advanced in a clinical trial of anti-MUC1-C CAR T cells and in IND-enabling studies for development as an antibody–drug conjugate (ADC). Agents targeting the MUC1-C cytoplasmic domain have also entered the clinic and are undergoing further development as candidates for advancing TNBC treatment. Eliminating TNBC CSCs will be necessary for curing this recalcitrant cancer and MUC1-C represents a promising druggable target for achieving that goal.

## 1. Background

### 1.1. Challenges in the Treatment of Advanced Triple-Negative Breast Cancer (TNBC)

Metastatic TNBC typically has a highly aggressive clinical course [1,2]. Cytotoxic chemotherapy remains the standard treatment for advanced disease [1]. However, in the metastatic setting, patients treated with platinum-based regimens have limited increases in progression-free survival as compared to those receiving other available options [3]. Germline *BRCA1*/*2* gene mutations, which occur in approximately 10% of patients, increase responsiveness to platinum-based agents [4]. In addition, TNBCs with biallelic *BRCA1/2* loss are dependent on PARP-mediated DNA repair and are sensitive to PARP inhibitors [5]. Despite these advances, patients invariably develop resistance to these agents and succumb to progressive disease [2].

Treatment of advanced TNBC with immune checkpoint inhibitors (ICIs) as monotherapy has also been associated with limited clinical benefit. Patients with PD-L1+ TNBCs and fewer previous lines of chemotherapy are more likely to respond to ICIs [2]. However, intrinsic resistance to ICIs occurs in 60–85% of patients [2]. Moreover, most patients who experience stable disease or objective responses develop acquired ICI resistance and few have lasting benefits [2]. Combining ICIs with chemotherapy has had more promising activity. Pembrolizumab in combination with chemotherapy has been approved as a first-line treatment for PD-L1+ advanced TNBC [6,7,8,9,10]. ICIs are also being combined with PARP inhibitors and antibody–drug conjugates (ADCs) for evaluation in clinical studies [2,11,12,13]. 

Major obstacles for the treatment of advanced TNBC are thus intrinsic and acquired resistance to genotoxic and immunotherapeutic agents. The cancer stem cell (CSC) state is associated with lineage plasticity, DNA damage resistance and immune evasion [14,15,16,17], indicating that elimination of CSCs will be necessary for achieving durable responses and cures. The effective treatment of TNBC will therefore clearly require the identification of druggable targets that are essential for the CSC state. 

### 1.2. Potential Therapeutic Targets of the TNBC CSC State

Recent articles have reviewed the importance of CSCs in TNBC progression and therapeutic resistance [18,19,20,21,22,23]. TNBCs are enriched with CD44+/CD24−/ALDH1+ CSCs that contribute to metastases, chemoresistance and poor clinical outcomes [24,25,26,27,28]. Resistance of TNBC CSCs to DNA damage is mediated by phenotypic heterogeneity and epithelial–mesenchymal plasticity, which are driven in part by the ZEB1 transcription factor (TF) [29,30,31,32]. The WNT, NF-κB and NOTCH pathways are activated in TNBC CSCs [20,33]. Activation of Sonic Hedgehog (SHH) has also been identified as a pathway that contributes to the TNBC CSC state [33,34,35]. Agents that target WNT, NF-κB, NOTCH and SHH signaling are under development to eliminate CSCs in TNBCs, as well as other cancers [20,21]. Along these lines, small molecule inhibitors of WNT/β-catenin/TCF signaling are being developed based on their inhibition of TNBC CSCs in cell line and tumor models [36,37,38]. Antibodies that inhibit the NOTCH pathway by blocking gamma-secretase-mediated cleavage and receptor–ligand interactions represent potential CSC-directed therapeutics [39]. In addition, a GLI inhibitor has been under investigation for targeting the SHH pathway in CSCs [40]. As another approach, a genomewide RNAi screen was used to identify potential therapeutic targets that control the BC CSC state, and this work led to the identification of salinomycin and JQ1 as agents with activity in TNBC PDX models [41]. Nonetheless, to our knowledge, these antibodies and signaling pathway inhibitors have not been advanced clinically as effective TNBC therapeutics.

Trophoblast cell surface antigen-2 (TROP-2) is a transmembrane glycoprotein that is overexpressed by TNBCs and other types of cancers [42,43]. TROP-2 plays a role in the self-renewal of stem cells and thus represents a potential target for TNBC CSCs [42,43]. Sacituzumab govitecan (SG; IMMU-1320) is an ADC that targets TROP-2 for the selective delivery of the irinotecan metabolite SN-38 [44]. SG is the first ADC approved for the treatment of TNBC based on activity in the advanced-disease setting [45,46,47,48]. In contrast to MMAE, which targets microtubules with cell cycle arrest, SN-38 is a topoisomerase I inhibitor that induces DNA damage [44]. The efficacy of SG is attributed to direct targeting of TROP-2-expressing TNBC cells, as well as the release of SN-38 into the TME with the killing of nontargeted cells [44]. 

Other approaches that inhibit signaling pathways shared by TNBC CSCs and non-CSCs, including PARP and CDK4/6 inhibitors [2,49], are under active development; however, what is needed, at least in part, are agents that selectively target TNBC CSCs. The present review summarizes compelling evidence that TNBC CSCs are addicted to the MUC1-C oncogenic protein, against which agents are under clinical and preclinical evaluation for TNBC treatment. 

### 1.3. Discovery of Mucin 1 (MUC1)

A monoclonal antibody, designated MAb DF3, was generated against a membrane-enriched fraction from a human breast carcinoma [50]. Studies with MAb DF3 led to the identification of the DF3 high molecular weight (>300 kD) protein. DF3 was found to be overexpressed on the entire cell surface and in the cytoplasm of breast cancer cells [50]. In contrast, DF3 was detectable at lower levels at the apical borders of normal breast epithelial cells [50]. MAbs generated against human milk fat globules (HMFG-1 and HMFG-2) were similarly found to recognize what was identified as the high molecular weight polymorphic epithelial membrane antigen (EMA, PEM) expressed on human breast cancer cells [51,52]. These early findings formed the basis for multiple studies of DF3 and PEM that explored their physical structures and expression patterns in normal and cancer cells. An advance in the field at that time was the discovery that the gene encoding these high molecular weight glycoproteins is frequently altered in breast cancers and contains conserved variable numbers of 60 base-pair tandem repeats (TRs) [53,54,55,56]. The encoded 20 amino acid TRs were found to be extensively modified by *O*-glycosylation, which identified a unique structure in a family of some 23 secreted and transmembrane mucins with the founding DF3/EMA/PEM member entitled mucin 1 (MUC1) [57]. These early findings collectively provided support for the potential importance of MUC1 as a cancer target [58]. Indeed, after a decade of fundamental and translational research, this progress in the field attracted interest in MUC1 as a potential target for developing anticancer vaccines and other therapeutic approaches [57,58]. 

### 1.4. Further Characterization of MUC1 as an Oncoprotein

**Figure 1:**
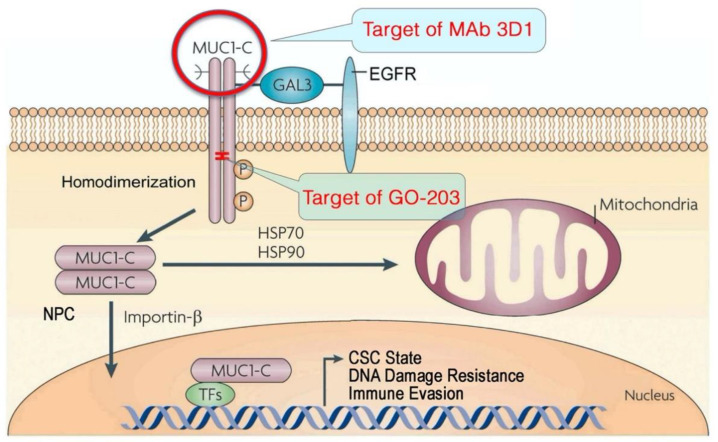
MUC1-C integrates activation of TNBC cell membrane signaling with the induction of EMT, lineage plasticity, DNA damage resistance and immune evasion. Activation of MUC1-C by loss of homeostasis is associated with the formation of homodimers that interact with EGFR and other RTKs at the cell membrane. MUC1-C homodimers are internalized by endocytic trafficking. Intracellular MUC1-C homodimers are transported to the mitochondrial outer membrane, where they block cell death. MUC1-C homodimers are also imported to the nucleus by importin-β and interactions with the nuclear pore complex (NPC). In the nucleus, MUC1-C interacts with TFs, such as WNT/β-catenin/TCF4, NF-κB, NOTCH and MYC, to drive EMT, epigenetic reprogramming and the cancer stem cell (CSC) state, which contribute to lineage plasticity, DNA damage resistance and immune evasion. Antibodies generated against the MUC1-C extracellular domain are being developed for CAR T cell and antibody–drug conjugate (ADC) immunotherapeutics. Targeting the MUC1-C cytoplasmic domain with the GO-203 inhibitor blocks MUC1-C homodimerization, nuclear import and function. Figure modified from [57,58].

MUC1 consists of two subunits that are formed by autoproteolysis of the translated protein Macao et al. 2006 [59]: (i) an extracellular *N*-terminal subunit (MUC1-N) with the glycosylated TRs that are characteristic of the mucin family and (ii) a transmembrane C-terminal subunit (MUC1-C) [57,58]. The MUC1-N/MUC1-C heterodimeric complex is expressed at the apical borders of normal epithelial cells, where it functions in protection against damage and inflammation (Figure 1) [57,58,60,61,62]. With loss of apical–basal polarity in response to disruption of homeostasis, MUC1-N is shed into a protective mucin barrier, where it forms complexes with pathogens that are then excreted by mucociliary transport. In addition, MUC1-C is expressed over the entire epithelial cell surface [58]. As a result, MUC1-C (i) interacts with receptor tyrosine kinases (RTKs) and other cell surface molecules normally positioned at the basal-lateral borders and (ii) integrates activation of cell membrane and intracellular signaling pathways (Figure 1).

MUC1-C consists of a 58-amino acid (aa) extracellular domain that is glycosylated on Asn-36, which then functions as a binding site for the galectin-3 ligand [63,64]. In turn, galectin-3 acts as a bridge to facilitate the association of MUC1-C with EGFR, FGFR, HER2 and other RTKs, and to activate their downstream signaling pathways (Figure 1) [63,64,65,66,67]. Activation of MUC1-C is associated with the formation of homodimers that are imported into the nucleus by importin-β and interactions with the nuclear pore complex [58,62,68]. In the nucleus, MUC1-C interacts directly with transcription factors, including STAT3 [69], WNT/β-catenin/TCF4 [70], NF-κB [71] and MYC [72], which as noted above have been linked to the CSC state, and contributes to the regulation of their target genes (Figure 1) [58,61]. MUC1-C is also transported by HSP70/HSP90 to the mitochondrial outer membrane, where it attenuates stress-induced apoptosis (Figure 1) [73,74].

The MUC1-C cytoplasmic domain consists of 72 aa, which include a CQC motif adjacent to the transmembrane domain that is necessary for the formation of homodimers and interactions with binding partners such as MYC [58,61,72]. Downstream of the CQC motif is an intrinsically disordered protein, as is often found in oncoproteins that integrate multiple signaling pathways [75,76]. 

### 1.5. MUC1-C Is of Importance to Hallmarks of TNBC Progression

MUC1-C contributes to HER2 activation and traztuzumab resistance in HER2+ breast cancer cells [67]. In addition, MUC1-C promotes tamoxifen resistance in ER+ breast cancer cells [77], indicating that it plays a significant role in different breast cancer subtypes. Here, we have focused on the importance of MUC1-C in the TNBC progression.

MUC1 is overexpressed in ~90% of TNBCs [58,61,78]. In concert with the capacity to intersect with diverse signaling pathways, MUC1-C is of importance to multiple hallmarks of breast cancer cells, including (i) increased growth [70], (ii) inhibition of death [79], (iii) metabolic alterations [80,81], (iv) induction of ZEB1, TWIST1 and EMT [82,83], (v) epigenetic reprogramming [84,85,86,87], (vi) chromatin remodeling [72,88], (vii) expression of stem cell markers [80,89] (viii) the CSC state, self-renewal capacity and tumorigenicity [83,90], (ix) genotoxic drug resistance [67,77,91,92,93] and (x) immune evasion [93,94,95].

Considerable evidence has emphasized the importance of lineage plasticity and the CSC state in driving therapeutic resistance [14,15,16,17]. In concert with that evidence, MUC1-C drives TNBC progression in association with induction of (i) EMT, (ii) self-renewal capacity, (iii) resistance to genotoxic agents and (iv) immune evasion [58]. These discoveries, which are summarized in more detail below, have collectively supported the addiction of TNBC CSCs to MUC1-C and have emphasized the importance of MUC1-C as a target for their elimination with agents developed against the MUC1-C extracellular and cytoplasmic domains.

### 1.6. TNBC CSCs Are Dependent on MUC1-C for Lineage Plasticity and Genotoxic Drug Resistance

MUC1-C interacts directly with the proinflammatory STAT3 and NF-κB TFs in cancer cells and contributes to the regulation of their target genes [58,61]. Involvement of MUC1-C in chronic activation of these pathways in cancer represents misappropriation of the role for MUC1-C in the protection of barrier epithelia from inflammation [57,58]. In TNBC cells, MUC1-C induces pSTAT3 and MUC1-C/pSTAT3 complexes that activate the *TWIST1* gene [83]. MUC1-C also interacts with the TWIST1 EMT-TF in driving (i) *MUC1* in an autoinductive circuit, (ii) ZEB1, SNAIL and other genes in the EMT program and (iii) the capacity for cell invasion. The MUC1-C/TWIST1 circuit also drives expression of the stem cell markers SOX2, BMI1, ALDH1 and CD44, self-renewal capacity and TNBC tumorigenicity. Consistent with the induction of lineage plasticity and the CSC state, MUC1-C and TWIST1 were shown to be necessary for acquired paclitaxel (PTX) resistance. MUC1-C drives luminal–basal dedifferentiation of TNBC cells by repressing *ESR1* and downstream ER pathways in association with the downregulation of FOXA1, GATA3 and other markers of the luminal phenotype. MUC1-C also represses expression of *BRCA1* [86], which plays a role in dictating mammary lineage stem cell fate [96]. In this way, MUC1-C integrates lineage plasticity, EMT and dedifferentiation in promoting the TNBC CSC state. Of translational relevance, targeting MUC1-C reverses PTX resistance as supported by synergistic activity with PTX against PTX-resistant TNBC cells. These findings uncovered dependence on MUC1-C for integrating lineage plasticity of TNBC CSCs with DNA damage resistance.

Mechanistic studies further demonstrated that MUC1-C activates ATM and promotes nuclear localization of the BMI1 and EZH2 polycomb group proteins, which form complexes with PARP1 in the DNA damage response (DDR) [92,97]. MUC1-C regulates (i) BMI1-induced H2A ubiquitylation, (ii) EZH2-driven H3K27 trimethylation and (iii) importantly, PARP1 activity. As a result, targeting MUC1-C sensitizes TNBC cells with mutant and wild-type BRCA1 to the PARP inhibitor olaparib. These findings (i) uncovered a role for MUC1-C in regulating PARP1 and promoting DNA damage resistance and (ii) identified a potential strategy for enhancing the effectiveness of taxanes, platinum-based agents and PARP inhibitors against TNBC CSCs.

Other mechanistic studies have demonstrated that MUC1-C promotes DNA damage resistance of TNBC CSCs by chronic activation of cytosolic nucleotide receptors and the cGAS-stimulator of IFN genes (STING) [93]. In TNBC CSCs, MUC1-C was found to be necessary for intrinsic expression of the RIG-I, MDA5 and cGAS cytosolic nucleotide pattern recognition receptors (PRRs) and STING. In concert with activating the PRR/STING axis, MUC1-C induces chronic IFN-β production and the inflammatory type I IFN pathway. As a result, MUC1-C induces the IFN-related DNA damage resistance gene signature (IRDS), which includes ISG15, in integrating chronic inflammation and DNA damage resistance. MUC1-C is also necessary for expression of PRRs, STING and ISG15 in the response of TNBC cells to treatment with carboplatin and olaparib, in further support of a critical role for MUC1-C in the DNA damage response (DDR). These findings and those with PTX demonstrated that MUC1-C plays a role in conferring intrinsic and acquired chemoresistance Rajabi et al. 2017 [98].

### 1.7. MUC1-C Promotes Immune Evasion of TNBC CSCs

The presence of tumor infiltrating lymphocytes (TILs), specifically CD8+ T cells, in the TNBC TME is associated with improved survival in response to chemotherapy [99,100,101]. The responsiveness of TNBCs to ICIs such as pembrolizumab is also improved by increases in TIL density [102,103,104,105,106], indicating that immune cell-depleted “cold” TMEs associate with resistance to treatment with genotoxic and immunotherapeutic agents. BRCA1/2 mutations and HR deficiency in TNBCs have not been associated with TIL density [107], indicating that factors other than mutational burden are of importance to depletion or dysfunction of TILs in the TNBC TME. CSCs have been linked to immune suppression across different types of cancer [15]. However, intrinsic effectors that contribute to the CSC state, “cold” TMEs and immune evasion remain largely known. 

MUC1-C activates the *PD-L1* gene in human TNBC cells by forming complexes with NF-κB on the *PD-L1* promoter region [94]. Targeting MUC1-C in an immunocompetent TNBC GEMM in which mouse Eo771/MUC1 cells are engrafted onto human MUC1.Tg mice suppresses PD-L1 expression in association with increases in CD8+ T cells and tumor-cell killing. Moreover, MUC1 expression in human TNBC tumors correlates inversely with CD8+ T cells in the TME and survival, providing evidence that MUC1-C may contribute to TNBC immune evasion. 

In support of those findings, subsequent studies demonstrated that MUC1-C activates the inflammatory type II IFN and JAK1→STAT1→IRF1 pathways in TNBC cells and tumors [95]. In this way, MUC1-C induces immunosuppressive effectors, including IDO1 and COX2/PTGS2, and associates with depletion and dysfunction of CD8+ T cells in the TNBC TME. These findings provided further evidence that MUC1-C activates immunosuppressive pathways in TNBCs and that MUC1-C is a potential target for inhibiting immune evasion of TNBC CSCs Rajabi et al. 2017 [98].

### 1.8. MUC1-C Is Necessary for the Remodeling of Chromatin in Driving the TNBC CSC State

Chromatin remodeling is critical for inducing the CSC state, lineage plasticity and therapeutic resistance. In concert with the dependence of TNBC CSCs on MUC1-C for DNA damage resistance and immune evasion, studies were performed to assess the effects of MUC1-C on reprogramming of chromatin to promote the CSC state. A previously unrecognized MUC1-C→MYC pathway was uncovered that regulates the NuRD chromatin remodeling and deacetylation complex [72]. A surprising discovery was that the MUC1-C cytoplasmic domain binds directly to the MYC HLH-LZ region, which also interacts with MAX. In turn, MUC1-C regulates the MYC transactivation function, which has opened a new area of investigation into involvement of MUC1-C in MYC signaling pathways. As one example, MUC1-C/MYC complexes activate the NuRD *MTA1* and *MBD3* genes in basal- and not luminal-type BC cells. In turn, MUC1-C recruits MTA1 and MBD3, as well as CHD4, to the *ESR1* promoter, represses ESR1 expression and attenuates downstream ER signaling. This MUC1-C→MYC→NuRD pathway also suppressed expression of FOXA1, GATA3 and other effectors of the luminal phenotype in driving dedifferentiation of TNBC CSCs. 

Other confirmatory work demonstrated that MUC1-C drives global changes in the chromatin architecture of TNBC CSCs [88]. MUC1-C induces differentially accessible regions (DARs) associated with differentially expressed genes (DEGs). Among these genes, we identified *NOTCH1*, which is required for TNBC cell self-renewal and the CSC state. MUC1-C activated *NOTCH1* by recruiting JUN/AP-1 and the BAF chromatin remodeling complex to a proximal enhancer-like signature in association with increases in chromatin accessibility. Similar results were obtained for the *EGR1* and *LY6E* stemness-associated genes, indicating that this MUC1-C-induced pathway of chromatin remodeling activates multiple genes that contribute to the TNBC CSC state. Taken together with regulation of the NuRD complex, these findings support an important role for MUC1-C in chromatin remodeling that drives luminal→basal dedifferentiation and lineage plasticity of TNBC CSCs [62].

These effects of MUC1-C on driving the TNBC CSC state extend to certain other cancers. In this regard, MUC1-C induces the Yamanaka pluripotency factors, EMT and self-renewal in pancreatic cancers [108] and castration-resistant prostate cancer (CRPC) [109]. In further support of the TNBC findings and consistent with driving the CSC state, MUC1-C also integrates lineage plasticity with chromatin remodeling, genotoxic drug resistance and immune evasion in these models [62,88,110,111,112].

### 1.9. Targeting MUC1-N for TNBC Treatment

Early attempts at targeting MUC1, particularly with antibodies, focused on MUC1-N and were ineffective in part because this subunit is shed from the cell surface [57,58,60,61]. In addition, the activity of antibodies generated against MUC1-N were limited by the large pool of circulating MUC1-N that has to be overcome for antibodies to reach the surface of cancer cells [58].

The provocative findings that the MUC1-N TRs are aberrantly under- or unglycosylated in cancer cells as compared to that in normal epithelia [113,114] invoked the possibility that vaccines and other immunotherapeutic approaches directed against the TRs could be developed to selectively treat human cancers. Indeed, after a decade of earlier work in the MUC1 field, groups of investigators became interested in studying preclinical and clinical anticancer vaccines for inducing immunity against unglycosylated TR peptides [115]. Despite this interest and the generation of anti-MUC1 TR immune responses, limited clinical benefit was observed in patients with cancer for advancing these vaccine approaches [116]. A notable example in this regard was the administration of the L-BLP25/Stimuvax/tecemotide vaccine, which induced an immune response against the MUC1-N TRs but was ineffective in prolonging overall survival in combination with chemoradiotherapy for patients with unresectable stage III NSCLC [58,117,118]. 

Aberrant *O*-glycosylation of the MUC1-N TRs in cancer results in truncated Tn and sialyl-Tn glycoforms that represent potential neoantigens for development of other immunotherapeutics [119]. Along these lines, anti-Tn-MUC1-N CAR-T cells developed by Dr. Carl June have entered a Phase 1 CART-TnMUC1-01 trial, which is now underway for therapy for patients with TnMUC1-positive advanced cancers (Tmunity Therapeutics).

### 1.10. MUC1-C Is a Target for Eliminating TNBC CSCs and Achieving Cures

In contrast to MUC1-N, the transmembrane MUC1-C subunit functions as an oncoprotein in TNBC CSC progression and is a potential druggable target for TNBC treatment [57,58,60,61,86,114]. A focus for advancing TNBC therapies has been the development of agents that target the MUC1-C 58 aa extracellular and 72 aa cytoplasmic domains [58]. 

**Figure 2:**
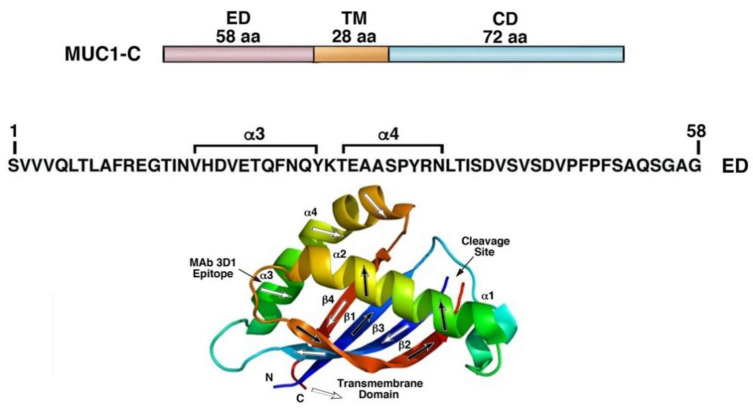
Targeting the MUC1-C extracellular domain. MUC1-C consists of a 58 aa extracellular domain (ED), 28 aa transmembrane domain (TM) and a 72 aa cytoplasmic domain (CD). The MUC1-C ED includes α3 and α4 helices that are exposed with MUC1-C activation and are targets for monoclonal antibodies (MAbs). MAb 3D1 has been generated against the α3 helix and is under development as CAR T cell and ADC approaches. Figure modified from [58,120].

MAb 3D1 was generated against the MUC1-C extracellular domain α-3 helix, which becomes exposed with MUC1-C activation [120] (Figure 2). Other antibodies are under development that target the junction between MUC1-N and MUC1-C [121,122] and the MUC1-C extracellular domain, including the SKM1 series [123] and MIN-C2 (Minerva Biotechnologies, Waltham, MA, USA). However, to our knowledge, there are no reports of MAbs that react with the α-3 helix.

The generation of MAb 3D1 against the MUC1-C extracellular domain provided an opportunity for the development of therapeutic agents, such as CAR T cells and ADCs, that target MUC1-C on the TNBC cell surface [120]. Along these lines, an anti-MUC1-C CAR T cell approach using MAb 3D1 sequences was highly effective against MDA-MB-468 TNBC tumor xenografts and has entered the clinic in a partnership with pharma for the treatment of TNBCs and other MUC1-C-expressing cancers (NCT05239143: P-MUC1C-ALLO1 Allogeneic CAR-T Cells in the Treatment of Subjects with Advanced or Metastatic Solid Tumors).

For the development of an ADC, MAb 3D1 was conjugated using a maleimidocaproyl-valine-citrulline-*p*-aminobenzyloxycarbonyl (vc) cleavable linker to the toxin monomethyl auristatin E (MMAE). The vc cleavable linker is widely used with MMAE in approved ADCs, such as Adcetris (Seattle Genetics, Bothell, WA, USA), Polivy (Roche Genentech, South San Francisco, CA, USA) and Padcev (Astellas, Tokyo, Japan), as well as in other ADCs under development [124,125,126]. 

MAb3D1-MMAE ADCs were highly potent in killing MUC1-C-expressing cancer cells in vitro with an IC50 in the nM range [115]. The MAb3D1-MMAE ADCs were also effective against established human tumor xenografts, including TNBC PDX models, without evidence of overt toxicity [120]. To confirm the absence of toxicity in NCG mice expressing mouse Muc1-C, toxicity studies were performed in human MUC1.Tg mice that express MUC1 at a level and pattern found in humans [127]. Studies in the MUC1.Tg model demonstrated no significant adverse effects of the MAb3D1-MMAE ADC on normal tissues or hematologic parameters [120]. These findings supported the notion that the MUC1-C α-3 helix is selectively exposed on the surface of carcinoma cells as compared to normal epithelia [120]. Based on this therapeutic selectivity, the anti-MUC1-C ADCs are now under development by the NCI NExT Program for IND, which is enabling studies and conducting early phase trials in patients with TNBCs. 

**Figure 3:**
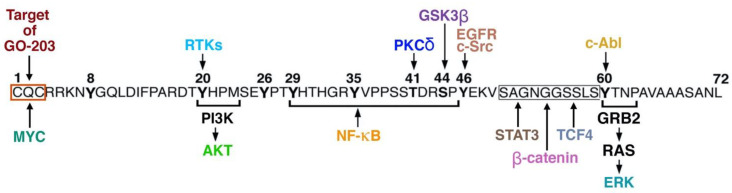
MUC1-C cytoplasmic domain interacts with diverse effectors of the CSC state. The MUC1-C CD (72 aa) is an intrinsically disordered protein that functions as a node in integrating multiple signaling pathways that promote the CSC state. MUC1-CD includes a CQC motif that is (i) a sensor of increases in ROS, (ii) necessary for the formation of MUC1-C homodimers and (iii) a target for the GO-203 inhibitor. The MUC1-CD CQC motif confers binding to MYC. Shown are tyrosine phosphorylation sites that function as motifs for interactions with PI3K (pYHPM) and GRB2 (pYTNP) in activating the AKT and ERK pathways, respectively. Also shown are potential consensus SH2 binding motifs for interactions with SHC (pYPTY) and PLCγ1 (pYVPP). In addition, MUC1-CD interacts directly with proinflammatory TFs, such as NF-κB and STAT3, and the WNT pathway effectors, β-catenin and TCF4, that are associated with driving the CSC state. Highlighted is the SAGNGGSSLS or SER-rich motif (SRM) that functions as a binding region for STAT3, β-catenin and TCF4. Figure modified from [57,98].

Other approaches have been under development for targeting the MUC1-C cytoplasmic domain (CD) [58]. MUC1-C CD is an intrinsically disordered protein devoid of kinase activity that includes a CQCRRK motif, which is necessary for MUC1-C homodimerization and function as an oncoprotein [58]. In this way, the MUC1-C cytoplasmic domain is phosphorylated by multiple kinases, such as RTKs, SRC, ABL and GSK3β, that in turn regulate direct interactions between MUC1-C and downstream effectors, including the MYC, NF-κB, STAT3 and WNT/β-catenin/TCF4 TFs, linked to the CSC state (Figure 3) [58,61,62].

Accordingly, the cell-penetrating GO-203 inhibitor (D-amino acids: (R9-CQCRRKN)) was developed to block the CQC motif and MUC1-C activity (Figure 3). In multiple studies, GO-203 exhibited dose-dependent activity against MUC1-expressing human tumor xenograft models [59,122,123,124,125]. In addition, GO-203 treatment phenocopies the effects of targeting MUC1-C genetically in cancer cells, supporting the use of this agent to inhibit MUC1-C function [58,108,109,128,129,130,131,132,133].

GO-203 is not a substrate for ABC transporters that are often upregulated in CSCs [58]. Along these lines, GO-203 is effective in inhibiting self-renewal capacity and tumorigenicity of human TNBC CSCs [58,72,83,92,133]. Consistent with resistance of TNBC CSCs to DNA damage, GO-203 is highly effective in potentiating the activity of genotoxic anticancer agents [58]. In studies of the Eo771/MUC1 GEMM TNBC, treatment with GO-203 also resulted in (i) downregulation of PD-L1 expression, (ii) activation of CD8+ T cells in the TME, (iii) induction of TILs effective in killing Eo771/MUC1 tumor cells and (iv) inhibition of Eo771/MUC1 tumor growth [94]. This TNBC GEMM thus has the potential for informing advances in the immunotherapy of TNBCs with anti-MUC1-C agents against the extracellular and cytoplasmic domains and for combination studies with immune-based therapies.

### 1.11. TNBC CSCs Are Addicted to MUC1-C for Lineage Plasticity and Therapeutic Resistance

In summary, TNBC CSCs are addicted to MUC1-C based on the definition that they are dependent on MUC1-C for survival [134]. TNBC CSCs are also dependent on MUC1-C for induction of EMT, epigenetic reprogramming and chromatin remodeling that are necessary for lineage plasticity, DNA damage resistance and immune evasion. These findings are of significance in having highlighted a critical role for MUC1-C in TNBC CSC progression and having emphasized the importance of developing agents that target the MUC1-C extracellular and cytoplasmic domains for TNBC treatment.
